# Bringing Virtual Reality From Clinical Trials to Clinical Practice for the Treatment of Eating Disorders: An Example Using Virtual Reality Cue Exposure Therapy

**DOI:** 10.2196/16386

**Published:** 2020-04-23

**Authors:** Theresa Brown, Emily Nauman Vogel, Sarah Adler, Cara Bohon, Kim Bullock, Katherine Nameth, Giuseppe Riva, Debra L Safer, Cristin D Runfola

**Affiliations:** 1 PGSP-Stanford PsyD Consortium Palo Alto, CA United States; 2 Department of Psychiatry and Behavioral Sciences Stanford University Stanford, CA United States; 3 Applied Technology for Neuro-Psychology Lab Istituto Auxologico Italiano Milan Italy; 4 Centro Studi e Ricerche di Psicologia della Comunicazione Università Cattolica del Sacro Cuore Milan Italy

**Keywords:** virtual reality, exposure therapy, eating disorders, translational research, technological innovation

## Abstract

Novel treatment options for eating disorders (EDs) are critically needed to enhance treatment outcomes and reduce the rates of treatment dropouts. On average, only 50% of individuals receiving evidence-based care remit, whereas 24% drop out before treatment completion. One particularly promising direction involves integrating virtual reality (VR) with existing evidence-based treatments (EBTs) such as cue exposure therapy (CET). Across psychiatric disorders, VR-based interventions are demonstrating at least preliminary efficacy and noninferiority to traditional treatments. Furthermore, VR technology has become increasingly portable, resulting in improved acceptance, increased access, and reductions in cost. However, more efficient research processes may be needed to uncover the potential benefits of these rapid technological advances. This viewpoint paper reviews existing empirical support for integrating VR with EBTs (with a focus on its use with EDs) and proposes key next steps to more rapidly bring this innovative technology-based intervention into real-world clinic settings, as warranted. VR-CET for EDs is used to illustrate a suggested process for developing such treatment enhancements. We recommend following a deployment-focused model of intervention development and testing to enable rapid implementation of robust, practice-ready treatments. In addition, our review highlights the need for a comprehensive clinical protocol that supports clinicians and researchers in the implementation and testing of VR-CET and identifies key missing protocol components with rationale for their inclusion. Ultimately, this work may lead to a more complete understanding of the full potential of the applications and integrations of VR into mental health care globally.

## Introduction

Eating disorders (EDs) pose serious risks to psychological and physical health [1,2], resulting in an elevated risk of death for those with EDs compared with the general population [3-5]. Although evidence-based treatments (EBTs) for EDs demonstrate reasonable efficacy, on average, only 50% of individuals receiving evidence-based care remit fully [6-8], and 24% are estimated to drop out before the completion of treatment [9]. Innovation is needed to improve treatment outcomes.

Virtual reality (VR) is a technology that can augment existing EBTs with the possibility of enhancing treatment outcomes. Through the creation of immersive computer-generated experiences, users interact naturally with stimuli representing the real world while simultaneously benefiting from a clinical, supervised setting [10]. Moreover, VR shares with the brain the same basic mechanism: embodied simulations [11]. In fact, a VR system, similar to the brain, maintains a model (simulation) of the body and the space around it.

These features offer different potential advantages for using VR to augment existing EBTs for EDs [12-16]. Advantages may include automating and standardizing ED psychoeducation, opportunities to practice emotion regulation skills, reprogramming attentional biases, enhancing insight by identifying body distortion, changing implicit and explicit perceptual bodily distortions, reducing weight stigma and biases, enhancing empathy among support persons, and augmenting exposure therapy. In particular, three different randomized controlled trials [14,17,18] have shown at long-term follow-ups (6 and 12 months) that VR-enhanced cognitive behavioral therapy (CBT) for EDs had a higher efficacy on some outcomes than CBT alone (eg, greater improvement in body image disturbances; increased reduction in the frequency of binge, purge, and overeating episodes; and more weight loss for obese individuals with binge eating disorder, BED).

Nevertheless, traditional models for testing treatments, which include case series and randomized controlled trials to test efficacy under highly controlled research conditions in academic centers, followed by effectiveness and implementation studies, may render these rapidly changing technologies obsolete by the time they are clinic-ready. For example, since the early 2000s, immersive VR has become increasingly sophisticated with improved 3-dimensional constructed environments; compatibility with mobile phones; and a wide variety of head-mounted display devices equipped with increased field of view, higher-resolution images, and lightweight and comfortable designs [19]. Testing these interventions within real-world clinic settings quickly, before they are replaced by newer technology, may be crucial to benefit from the existing content and devices [20].

In this paper, we discuss the great potential of VR-based interventions in the treatment of psychiatric disorders and research efforts needed to support the development of effective clinic-ready treatments in a timely manner. Of the many applications of VR in the treatment of EDs [21,22], virtual reality cue exposure therapy (VR-CET) has the strongest empirical support at present [18,23-25]. Therefore, we focus on VR-CET to anchor the discussion. Specifically, this paper provides: (1) an overview of VR-CET and its potential advantages to augment EBTs; (2) a brief review of the empirical support for VR-based exposures in psychiatric disorders, with a focus on VR-CET for EDs; (3) an argument for small-scale effectiveness trials early in treatment development (following the deployment-focused model of intervention development and testing by Weisz et al [20]); and (4) ideas on how VR-CET can be more rapidly developed and implemented in real-world clinic settings, highlighting key missing protocol components, with rationale for their inclusion.

## Advantages of Virtual Reality–Cue Exposure Therapy

VR-CET provides traditional cue exposure therapy (CET) in a virtual environment. CET is a subcategory of exposure therapy that is specific to eating and substance-related disorders. Both techniques involve repeated, controlled exposures to relevant stimuli. However, CET was initially specifically designed to target cravings (vs fear) [26]. CET is based on classical conditioning, a learning theory that explains how maladaptive behavior can develop in response to previously neutral stimuli. According to classical conditioning, repeatedly pairing a neutral stimulus (conditioned stimuli) with a stimulus (unconditioned stimulus) that naturally evokes a biologically potent response (unconditioned response) may eventually result in responding similarly to the neutral stimulus as one would for the biologically potent stimulus (conditioned response). For example, in the classical conditioning model of binge eating, Jansen [27] conceptualizes the intake of food as the unconditioned stimulus and its metabolic effects as the unconditioned response. Cues that reliably signal food intake, such as sight, smell, taste, and the context in which one eats, act as conditioned stimuli. The presence of these cues (conditioned stimuli) elicits physiological responses that are experienced as craving (ie, an almost irresistible urge to eat), which can increase the probability of binge episodes (conditioned response) [28-31]. In CET, the main objective is to weaken the bond between the cues (conditioned stimuli) and the maladaptive responses (conditioned responses), which may mask or inhibit initial learning [32]. Specifically, VR can reduce eating-related anxiety during and after exposure to virtual food, helping to disrupt the reconsolidation of adverse, food-related memories [24,26].

In VR-CET for EDs, patients are repeatedly exposed to emotionally provoking eating-related situations that typically result in maladaptive behaviors (eg, binge eating and avoidance). Exposure is planned gradually and designed to eliminate the ED behavior. New associations develop with eating-related anxiety and cravings in response to stimuli decreasing over time [24,33]. Emotional changes may occur, in part, because of the modifications to dysfunctional thinking and increased self-efficacy [34]. Other VR exposure programs created in the context of CBT extend the exposure activities to include cognitive restructuring and practice using alternative emotion regulation strategies in response to triggering stimuli. These strategies may help address core ED symptomatology [31].

VR offers several promising advantages to in vivo exposure that may result in reduced therapist burden, more rapid symptom improvement, improved acceptance of treatment, reduced treatment dropouts, and more accurate measurement-based care. VR environments may more closely approximate the settings in which problematic eating behaviors take place compared with the clinician’s office or imaginal exercises. A therapist can manipulate a larger number of stimuli within the VR environment than in the real world (eg, intensity of the feared stimulus) and personalize both contextual cues (eg, social setting and room type) and sensorial cues (eg, smell, sound, and tactile effects). VR environments can be tailored precisely to a patient’s fear hierarchy (ie, the client’s rank order list of cues that elicit anxiety on a scale from lowest to highest). Extensive evidence exists showing VR environments produce emotional, behavioral, and physiological responses in patients similar to those observed in real-life situations [15]. Thus, VR enables the therapist to guide the patient through an exposure or use skills within a more ecologically valid environment, efficiently challenging dysfunctional mental representations [34] and potentially facilitating improved application of the acquired skills and subsequent generalization. In addition, VR-based exposures may be a more acceptable strategy than the traditional leap from an in-office imaginal exposure to an in vivo exposure. Gradually moving from virtual to in vivo exposures offers a middle step and a more palliative intervention that may reduce treatment dropout rates [31,33]. The appeal of a new and exciting technology may also contribute to the patients’ willingness to engage with exposures in VR. Indeed, one study found that 89% of patients with specific phobia chose VR over in vivo exposures when given a choice [35]. Preference for VR over in vivo exposures may very well be a manifestation of avoidance of the feared stimuli in patients with specific phobia. For example, about 25% of patients with specific phobia refuse exposure therapy [35]. However, reviews [36,37] show limited evidence that safety behaviors may not be detrimental to exposure efficacy based on the inhibitory learning model and thus conclude that judicious use of safety behavior can be clinically indicated. It is recommended to eliminate safety behavior as soon as the clients are willing. Thus, in cases in which avoidance appears to drive the patient’s rationale for in virtuo exposure, VR may serve as a stepping-stone to in vivo exposure for patients who would not otherwise receive care. Furthermore, a review of VR in the treatment of EDs indicated increased motivation for change in VR treatments and lower rates of loss to follow-up compared with in vivo active comparisons across several studies [10]. Finally, data collection and measurements can occur in session more effectively when using VR. This can eliminate potential adherence and validity problems with in vivo exposure compliance tracking. VR can enable tracking of many ecologically valid variables (eg, level of anxiety and urge to binge), which can be recorded and viewed in real time [38].

Unfortunately, studies indicate that many therapists hold negative beliefs about in vivo exposure therapy for anxiety disorders (eg, exposure may harm patients, result in symptom exacerbation, or harm therapists because of vicarious trauma), which may pose a barrier to treatment dissemination [39]. In contrast, there is growing interest and increasing acceptability among therapists regarding VR-based exposure. For instance, recent studies suggest that therapists have an overall positive attitude toward VR exposure therapy (pros rated higher than cons) and view VR as applicable to conditions other than anxiety [40], including EDs [41]. Lindner et al [40] also noted that high financial costs and technical difficulties were no longer top-rated negative aspects with the release of consumer VR platforms. However, the authors note that VR has not yet been widely implemented in routine care, for unclear reasons.

## Empirical Support for Virtual Reality–Based Exposure With a Focus on Eating Disorders

The intention of this paper is not to systematically review the literature on VR-based exposure therapy because that would be outside the scope of this viewpoint paper. Instead, we provide an overview of the empirical support for VR-based exposure with a focus on EDs to highlight the foundational work and provide context for our arguments in the subsequent sections. A substantial body of research supports the efficacy of VR-CET and VR-based exposures for numerous psychiatric disorders, notably those that frequently co-occur with EDs such as anxiety, substance use disorders, and posttraumatic stress disorder (PTSD) [42-44]. VR exposure therapy is consistently more effective than nonactive control groups (ie, waitlist), whereas comparisons between VR exposure therapy and manualized CBT and/or in vivo exposures tend to show equivalence in outcomes [43-49]. Considerable research has focused on anxiety disorders (eg, social phobia, panic disorder with agoraphobia, and specific phobias), with meta-analytic studies reporting large effect sizes (eg, Cohen *d*=0.95, ranging from 0.87 in PTSD to 1.79 in panic disorder with agoraphobia) [50]. Meta-analyses also reveal moderate effect sizes for VR exposure therapy over waitlist controls in PTSD [48,49] and show the benefit of VR exposure therapy in the assessment or treatment of acute stress disorder and paranoia [51]. These reviews note that although more research is needed to better understand the mechanisms of action, VR-specific variables such as a sense of presence within virtual environments may affect treatment outcomes [43,45]. Additional important takeaways are that modern VR systems are becoming increasingly affordable, accessible, and user-friendly [44] and that VR may be especially useful in exploring hypotheses related to the processes and mechanisms involved in exposure therapy because of the high degree of control and manipulation of specific variables that this technology allows [45]. Finally, the existing reviews illustrate the insufficient state of the current literature, given inconsistent reporting of key variables [43] and the fact that although studies have been conducted in controlled research contexts, research within real-world clinical settings are lacking [45,51].

Compared with research on the use of VR-based exposures for anxiety and related disorders, the number of studies that have specifically investigated the use of VR-CET for EDs is small. With so few individual studies conducted to date, publication of meta-analyses or reviews of VR-CET for EDs has not yet been warranted. However, the limited research conducted thus far is favorable. For example, patients with bulimia nervosa (BN) and BED who remained symptomatic after CBT showed a significantly greater reduction in binge eating and higher percentages of abstinence from binge eating and purging after randomization to six sessions of VR-CET vs six sessions of additional CBT [23]. Results from a 6-month follow-up of this same study revealed that reductions in binge, purge, and overeating episodes were greater after treatment with VR-CET [18]. Such results suggest VR-CET is not only efficacious posttreatment but appears to have lasting effects.

A total of three case reports using a similar nonimmersive VR-CET program as a complementary tool to CBT demonstrated positive effects with patients diagnosed with restrictive anorexia nervosa (AN-R), binge/purge anorexia nervosa (AN-B/P), and BN [52-54]. All patients reported lower levels of anxiety as well as reduced frequency of safety and avoidance behaviors related to food after completing a short (six or seven sessions) VR-CET module. Notably, the patient diagnosed with AN-R increased her BMI from 15 kg/m^2^ to 16.8 kg/m^2^ [52], the patient diagnosed with AN-B/P reduced her binge/purge behaviors from two to three times per day to once per week [53], and the patient diagnosed with BN completely eliminated binge and purge episodes [54]. Furthermore, the patients in these case reports reported that VR treatment was acceptable and helpful [52-54]. Of note, VR exposure was particularly helpful for exploring thoughts and emotions experienced in the moment while *eating* a virtual food, enabling productive therapeutic discussions to take place in an “‘ecological’ environment, one that was clinically significant but also safe” [53].

Given the aforementioned research, VR-CET for EDs may have value for advancing the field of EDs. The patients’ responses to VR-CET outlined in the research above are rapid. Faster acting treatments may reduce treatment length and, as a result, reduce clinic wait times—thereby positively addressing access to care issues. Given these potential advantages, we discuss the importance of pilot testing in real-world clinical settings at this stage in the developmental process.

## Importance of Small-Scale Effectiveness Trials

A recent systematic review of VR in the assessment, understanding, and treatment of mental health disorders broadly argues that the progress in implementing and disseminating VR applications has been slow despite growing interest [55]. The authors propose that VR in mental health care could be *revolutionary* in that the results of VR treatment may surpass those of a standard course of EBT [55]. Reilly et al [56] specifically argue for the expansion of exposure approaches and techniques, including VR-CET, for EDs. However, to date, the studies employing VR-CET for EDs have been conducted in Europe under the highly controlled settings of research trials. To translate this research into clinical practice, VR-CET needs to be tested in real-world clinic settings.

Historically, across disorders, implementation of VR in treatment was limited by the cost of technology and concerns about acceptability (eg, motion sickness experienced by a high percentage of participants) [57,58]. However, over time, VR technology has become increasingly portable (with the advent of handheld devices), resulting in improved acceptance, increased access, and reduction in cost. Market trends suggest that the demand for VR will rise steeply, with estimates of 55 million headset orders for 2022 alone [59], making VR nearly ubiquitous in American homes, similar to the personal computer. This increased access increases the likelihood of bringing VR-CET into the clinic, with supporting use at home. Investigation of how best to translate the existing research into real-world application is timely.

Given the limited number of efficacy trials of VR-CET for EDs to date, some may argue that translating this treatment into clinical practice at this stage of development is premature. In traditional models of EBT development, efficacy trials that provide substantial evidence for the treatment’s success under controlled conditions are conducted before studying the treatment in real-world settings. However, as other researchers have observed [20], the gap between research and real-world conditions is sometimes so big that regardless of the treatment’s robustness under controlled conditions, the treatment is not able to endure the conditions of the real world; the task of closing this gap at the end of a series of efficacy trials becomes unnecessarily complex and inefficient. As the former National Institute of Mental Health (NIMH) Director Thomas Insel [60] pointed out, interventions developed in highly controlled research settings often fail to take into account the key characteristics of the patients, providers, and settings in which the intervention will ultimately be implemented, which limits their practical value. For example, patients with comorbid issues are often excluded, and interventions are conducted by highly trained and closely supervised clinicians. However, in practice settings, comorbid issues tend to be the rule rather than the exception, and up to 40% of mental health providers in the public sector do not have a graduate or professional degree [60]. In addition, setting factors such as billing constraints and typical patterns of patient service use (eg, number of sessions typically attended in the setting) tend to be overlooked. As a result, treatments developed under highly controlled settings may be scientifically valid but not necessarily clinically meaningful or generalizable to real-world clinic settings [60].

As an alternative to traditional models of treatment development, Weisz et al [20] describe a “deployment-focused model of intervention development and testing” that integrates testing of treatments in practice settings early and throughout the treatment development process, rather than as a final phase, to ensure that they are applicable to and successful in the settings in which they will be delivered. Notably, the NIMH also shows interest in supporting clinic-based treatment development approaches in the NIMH Strategic Plan for Research [61]. We argue that deployment-focused model is not only particularly applicable to VR-CET but also an integral component of its successful development and testing—particularly given the likelihood of the technologies involved to become obsolete. Estimates of the time lag between when a treatment is initially developed and when it reaches the stage of dissemination, following the traditional research models, average about 17 years [62]. We thus identify a next direction for researchers and practitioners to translate this research into practice.

## Translating Virtual Reality–Based Clinical Trials to Clinical Practice: Future Directions

Weisz et al [20] propose that the first stage of deployment-focused treatment development involves the creation of a treatment protocol. Indeed, bringing VR-CET into clinical practice at this stage of development requires the creation of a protocol that is easily disseminated; adaptable across cultures and settings; and clearly outlines acceptability, feasibility, and implementation factors. As such, characteristics of providers and the usual settings in which care is provided must be considered. Many factors that would inform the creation of this protocol and ensure that VR-CET is practice-ready are noted in the literature as important but not consistently reported or described in sufficient detail. More comprehensive and consistent reporting of these factors has the potential to inform a VR-CET protocol that would support researchers and clinicians in becoming comfortable in implementing a technological innovation that has potential to advance the treatment for EDs. These factors include (1) patient and clinician reactions to and satisfaction with VR technology [40,63]; (2) logistical parameters such as space required, costs, and institutional buy-in [63]; (3) considerations in creating VR content, including working with software and/or third-party vendors and the optimal level of *presence* (the extent to which the individual interacts with the VR environment as if it is reality) for treatment effects to occur [64,65]; (4) clinical guidelines and outcome data, including outcomes compared with traditional EBTs, how to determine patient suitability for VR, optimal frequency and number of sessions, and the amount of time within the VR environment [66-68]; and (5) parameters regarding safety and acceptability, including cybersickness (side effects resulting from our physiological motion detection systems when in VR) [66]. Addressing these factors in a treatment protocol and providing examples for how to successfully execute each point will help move VR-CET from research settings to clinical practice.

In addition, future research should take advantage of the unique methods of data collection and assessment available within VR, including the collection of real-time self-reported data in virtuo and biological measurements that can track eye movements, facial gestures, and the movement of body parts. Given the large number of stimuli that can be manipulated and tightly controlled within VR environments (eg, intensity of stimuli, contextual, and sensorial cues), basic science research can utilize VR applications to advance the understanding of ED mechanisms of change.

Given that we are suggesting a deployment-focused model of treatment development for a novel iteration (VR-based) of an already validated therapy (CET), it is important to consider the risks compared with potential benefits of treatment implementation. One important risk associated with the utilization of any VR platform includes cybersickness, a side effect that 20% to 80% of VR users may experience [69,70]. The symptoms of cybersickness are similar to motion sickness and can include nausea, headaches, and dizziness [71]. For most people, cybersickness occurs about 15 min into the VR immersion, is worst in the first session, and becomes negligible by the third session [66]. In addition, clear procedures to address cybersickness within VR-based exposure protocols can mitigate the risks associated with these unpleasant symptoms and should always be included when implementing VR-based therapies [66]. Another risk is the potential loss of effectiveness when translating an already validated therapy into VR-based therapy. A recent review examined the negative effects of VR-based treatments for anxiety disorders using deterioration rates (rates of worsening symptomatology) as the primary outcome [72]. Deterioration rates for VR were found to coincide with other therapeutic approaches; the authors concluded that VR appears to be a nondeleterious treatment for patients with anxiety disorders [72]. In addition, meta-analytic studies show that VR exposure therapy for anxiety-related and trauma-related or stress-related disorders is not significantly less effective than in vivo treatment [43-49]*.*

Overall, the evidence thus far suggests minimal known risk associated with VR exposure therapy, which may reduce concern for deployment-focused treatment development. In addition, the standard of care in clinical practice involves the use of an informed consent process by which patients are provided with clear information regarding their treatment options, including the relative risks and potential benefits of each approach. Given the minimal known risks associated with VR-based treatments and the ability of VR-based exposures to provide an acceptable and effective treatment option for those who refuse, drop out of, or do not progress via in vivo exposures, we believe the potential benefits outweigh the costs of offering VR-based exposures as an alternative treatment option. To further minimize any associated risks of offering a novel treatment alternative to an already validated treatment approach, we suggest that the real-world trials of this technology should assess the patients’ progress and suggest in vivo exposures or a different active treatment if a patient is not making progress.

Given evidence that integrating VR technology with EBTs for EDs such as CBT leads to significantly improved outcomes [12,15], with faster effects and better maintenance than standard treatment alone [12,16], following the above recommendations to create and implement a comprehensive VR-CET protocol may help make evidence-based care more accessible and cost-effective for patients with an ED. We hypothesize that implementing VR-CET in real-world clinical settings may also foster increased patient participation in and excitement about treatment through their involvement in designing VR-based exposures. Furthermore, a patient’s experience of success (eg, tolerating the urge to binge) within VR may increase their perception of self-efficacy, enhancing confidence to translate therapy skills to real-world experiences.

Overall, comprehensive study of VR-CET in real-world clinic settings is a fruitful direction that may advance intervention protocols for EDs. In particular, a clear protocol for VR-CET for EDs will help translate the exciting research supporting the applications of VR into a clinic-ready intervention, providing a model use and, ultimately, a more comprehensive understanding of the full potential of the applications of VR on mental health care globally. We suggest that continued research efforts focus on advancing VR-CET following the clinic-based treatment development approach to more rapidly move technology-based interventions from research settings into the real world, as warranted.

## Abbreviations

AN-B/P: anorexia nervosa, binge/purge type
AN-R: anorexia nervosa, restrictive type
BED: binge eating disorder
BN: bulimia nervosa
CBT: cognitive behavioral therapy
CET: cue exposure therapy
EBT: evidence-based treatment
ED: eating disorder
NIMH: National Institute of Mental Health
PTSD: posttraumatic stress disorder
VR: virtual reality
VR-CET: virtual reality cue exposure therapy
